# Therapeutic Effect of *Ilex hainanensis* Merr. Extract on Essential Hypertension: A Systematic Review and Meta-Analysis of Randomized Controlled Trials

**DOI:** 10.3389/fphar.2018.00424

**Published:** 2018-05-08

**Authors:** Xiaochen Yang, Guoyan Yang, Weina Li, Yun Zhang, Jie Wang

**Affiliations:** ^1^Department of Cardiology, Guang'anmen Hospital, China Academy of Chinese Medical Sciences, Beijing, China; ^2^Centre for Complementary Medicine Research, University of Western Sydney, Sydney, NSW, Australia

**Keywords:** *Ilex hainanensis* Merr., hypertension, traditional Chinese medicine, systematic review, meta-analysis

## Abstract

With a rapidly aging population, the prevalence of hypertension in adults continues to rise, placing a substantial and escalating social and economic burden. *Ilex hainanensis* Merr. is commonly used as a folk remedy for treating hypertension, dyslipidemia, and inflammation in China. This systematic review aims to evaluate current evidence for the therapeutic effect of *Ilex hainanensis* Merr. extract (EIH) on essential hypertension. Six electronic databases (Pubmed, MEDLINE, The Cochrane Central Register of Controlled Trials, Chinese Scientific Journals Database, Wanfang and CNKI) were searched to identify eligible randomized controlled trials (RCTs) relevant to EIH on essential hypertension up to Jan 2018. Six RCTs including 772 participants met eligibility criteria. Methodological quality of the trials was generally low. Meta-analysis showed that EIH demonstrated a beneficial effect for lowering systolic and diastolic blood pressure (SBP/DBP), left ventricular mass (LVM) in participants with essential hypertension. There was no significant difference between EIH and antihypertensive drugs in SBP (WMD: −0.44 [−2.30, 1.43]; *P* = 0.65), DBP (WMD: WMD: −0.02 [−1.13, 1.09]; *P* = 0.98) and LVM (WMD: −1.36 [−4.99, 2.26]; *P* = 0.46). Moreover, one trial showed that EIH combined with antihypertensive drugs was more effective in lowering blood pressure than those antihypertensive drugs used alone. However, the findings were limited by the small sample sizes, duration and low methodological quality of the trials. This is the first systematic review of EIH on essential hypertension. More rigorous RCTs with high quality are still needed to prove the effectiveness and safety of EIH and its preparations for essential hypertension.

## Introduction

Hypertension is one of the leading causes of death and disability-adjusted life years worldwide. With a rapidly aging population, the prevalence of hypertension and related cardiovascular morbidity continues to rise, placing a substantial and escalating social and economic burden (NCD-RisC, 2017). In the United States, hypertension accounted for more cardiovascular diseases (CVD) deaths than any other modifiable CVD risk factor (Ford, 2011). According to a follow-up study of NHANES (National Health and Nutrition Examination Survey, 23,272 participants), more than half of deaths from coronary heart disease (CHD) and stroke occurred among individuals with hypertension. The risk of cardiovascular diseases is also significantly increased with uncontrolled blood pressure (BP) in China (Yang et al., 2007; Kario, 2013). The prevalence of hypertension in Chinese patients is 39% overall (Sheng et al., 2013), 59.4% in patients aged ≥ 60 years and 72.8% in those aged ≥ 75 years (Sheng et al., 2013). High blood pressure should be treated earlier with lifestyle changes and in some patients with medication at 130/80 millimeters of mercury (mmHg) rather than 140/90 mmHg based on the American Heart Association (AHA) guidelines for the detection, prevention, management and treatment of high blood pressure in November 2017. Therefore, promising new treatments to slow or stop the progress of hypertension are urgently needed.

**Figure 1:**
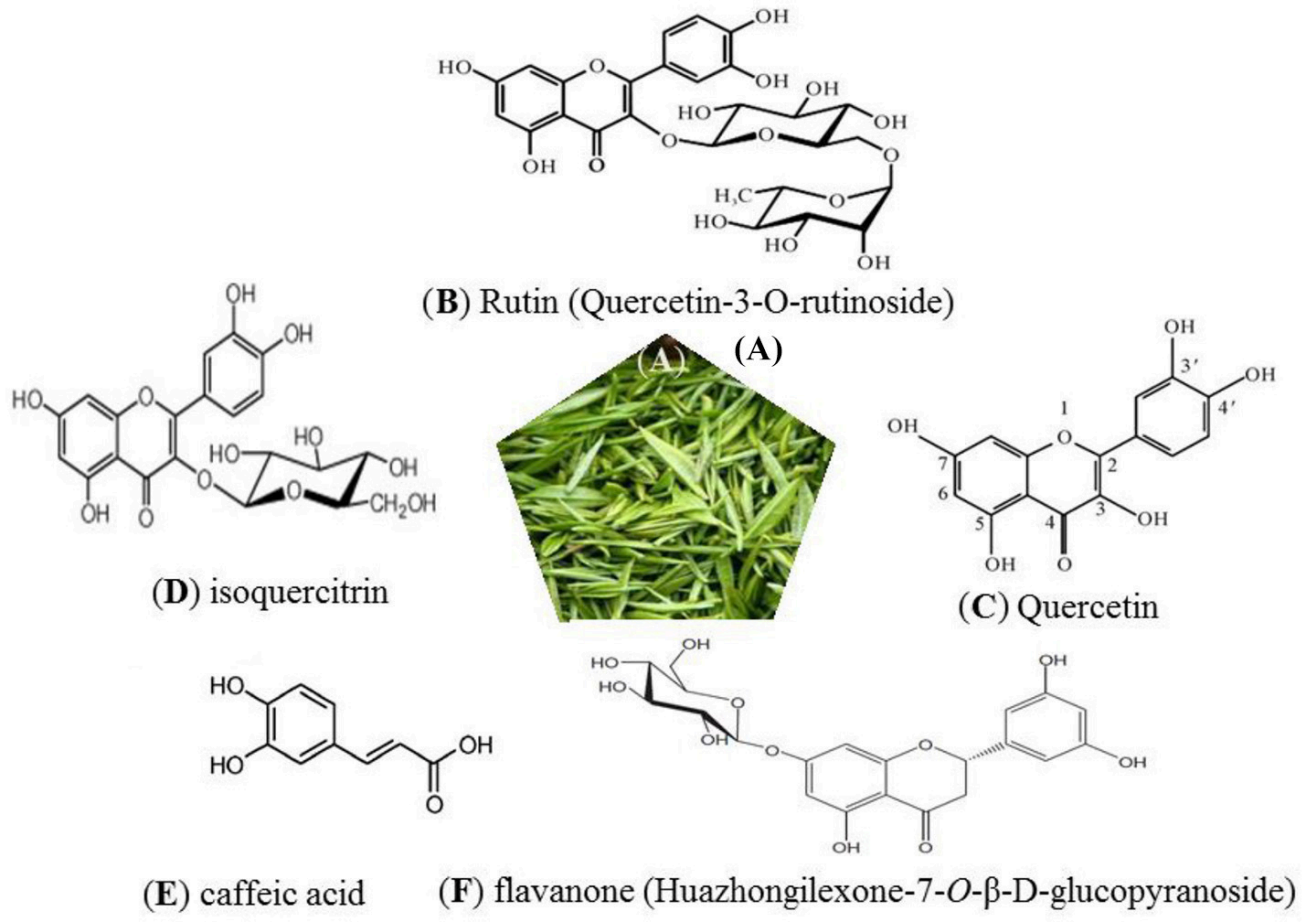
Leaves of *Ilex hainanensis* Merr., known as “Shan-Lv-Cha” **(A)**. Structure of several known constituents: rutin **(B)**, quercetin **(C)**, isoquercitrin **(D)**, caffeic acid **(E)**, and flavanone **(F)**.

Several lines of studies have indicated that traditional Chinese medicine (TCM) can be important modulators in the prevention of a variety of chronic diseases, because of their special characteristics such as multi-ingredient, multi-target, and less side effects (Yang et al., 2014; Xiong et al., 2017). *Ilex hainanensis* Merr., distributed mainly in the southern region of China, is used as an agent for resolving edema and relieving pain, invigorating blood and unblocking the collaterals according to TCM theory (Ernst, 2000). Its leaves, known as Shan-Lv medicine, have been used as a traditional tea product for relieving symptoms such as headache, dizziness and tinnitus, brain swelling, upset, irritability, and insomnia, which particularly related to hypertension in modern western medicine. Its therapeutic effects are not only related to antihypertensive, but also antilipemic, cholesterol-lowering, and anti-inflammatory, etc. In clinical practice, it has also been used for treating numerous chronic diseases such as coronary heart disease, cerebrovascular disease, nonalcoholic fatty liver disease, etc. (Yin et al., 2015). The extract of *I. hainanensis* (EIH) has been used in three preparations: shan_lv_cha antihypertensive capsule, jue_ming_shan_lv_cha tablet, and shan_lv_cha antihypertensive tablet, which has been included in the Pharmacopeia of the People's Republic of China in 2010. At present, researches on chemical composition and pharmacological action of EIH have been studied. A variety of compounds, including flavonoids, caffeoylquinic acid, triterpene acids, triterpenoid saponins, essential oil, organic acids have been isolated and identified (Markham and Ternai, 1976; Hang and Cao, 1984; Zhou et al., 2007; Chena et al., 2009; Chen et al., 2009; Cui et al., 2013; Yang et al., 2013) (Figure 1). Anti-hypertensive properties of EIH have been studied both *in vitro* and *in vivo*. Liu et al. (2010), for example, showed that EIH was capable of lowering blood pressure in murine model. The result demonstrated that the systolic blood pressure of EIH group (ten rats) decreased from 15.5 ± 1.8 KPa to 11.8 ± 2.8 KPa after 6-week treatment, compared with control group (10 rats treated with saline solution) decreased from 15.2 ± 1.7 KPa to 15.0 ± 1.7 KPa. EIH treatment also could significantly reduce the spontaneous activity of rats and increase the tendency of sleepiness. Another group (Li et al., 2008) also reported that EIH via duodenal administration was able to blood pressure in anesthetized cats. There was no significant difference between EIH group and captopril group after 2-h treatment. The active ingredient of its antihypertensive effect may be the Rutin in EIH. Another ingredient from flex hainanensis Merr., the triterpenoid-rich fraction (TF) had potential ability to protect liver against non-alcoholic fatty liver disease (NAFLD) by regulating lipids metabolism and alleviating insulin resistance, inflammation and oxidative stress in NAFLD murine model. The possible mechanism may be associated with regulating PPARα and CYP2El expression (Cui et al., 2013). In Yang's study (Yang et al., 2013), a sensitive and selective LC–MS method was developed to simutaneously determine chlorogenic acid, kaempferol-7-*O*-β-D-glucoside and ilexgenin A in rat plasma. The results revealed the pharmacokinetic behaviors of chlorogenic acid, kaempferol-7-*O*-β-D-glucoside, and ilexgenin A could be significantly changed in NAFLD rats after oral administration of EIH compared with normal rats. *In vitro* study (Sun et al., 2017), Ilexgenin A (IA), a novel pentacyclic triterpenoid, which extracted from leaves of EIH, could significantly inhibit ERK 1/2 phosphorylation in RAW 264.7 cells induced by LPS. The results demonstrated that IA might as an anti-inflammatory agent candidate for inflammatory disease therapy. Although EIH has been reported to have a broad range of pharmacological effects, including blood pressure-lowering, cholesterol-lowering, and anti-inflammatory, the underlying mechanism of many ingredients in EIH is still unclear.

Numerous clinical studies also showed that EIH could help to control blood pressure (Yang et al., 2000; Hui and Shu, 2002; Liu, 2002; Tang et al., 2004; Yang, 2006; Su and Guo, 2015). It is a folk remedy to treat patients with essential hypertension with EIH alone or combined with antihypertensive agents as an alternative method in south China. However, the evidence examining the effectiveness of EIH for essential hypertension has never been systematically summarized. Thus, this is the first systematic review to critically assess the effectiveness of EIH for essential hypertension.

## Methodology

### Study selection criteria

Randomized control trials (RCTs) published in Chinese or English until Jan 2018 on comparing EIH with western antihypertensive drugs or placebo in treating essential hypertension were screened. Cross-over randomized trials were included, but only the outcomes from the first period of treatment were extracted and analyzed. Quasi-randomized trials were excluded. Animal studies, clinical trials including case report, case series traditional reviews were also excluded.

Inclusion criteria were as follow: (a) participants with essential hypertension (SBP/DBP>140/90 mmHg); (b) the study was claimed to be an RCT; (c) the study compared EIH with conventional intervention or placebo or no treatment were included. RCTs comparing EIH combined antihypertensive drugs with antihypertensive drugs were also included; (d) duration of treatment (follow-up period) was at least 2 weeks.

Exclusion criteria were as follow: (a) duplicated or redundant study; (b) EIH was used in combination with other drugs; (c) the study did not include systolic blood pressure or diastolic blood pressure as the major outcome. (d) Participants with serious medical conditions, such as severe heart failure, dilated cardiomyopathy, and hypertensive encephalopathy.

### Search strategy

Two authors (YXC and YGY) searched the following electronic databases until January, 2018: Pubmed, Medline, China Network Knowledge Infrastructure (CNKI), Chinese Scientific Journals Database (VIP), Wan Fang Database, Cochrane Dementia and Cognitive Improvement Group and Cochrane Central Register of Controlled Trials (CENTRAL) in the Cochrane Library (January, 2018). The unpublished postgraduate theses in Chinese databases were also searched and the reference lists of all relevant papers found electronically were hand-searched.

The English searching terms were used individually or combined including “*Ilex hainanensis* Merr.,” “placebo,” “blood pressure,” “Essential Hypertension,” “Primary Hypertension,” “randomized controlled trial,” “controlled clinical trial,” “randomization,” “randomly,” “trial,” and “randomized.” The Chinese searching terms were used individually or combined including those for the generic name of Ilex hainanensis Merr. (“hai_nan_dong_qing”), trade names for Ilex hainanensis Merr. (“shan_lv_cha,” “shan_lv_cha_jiang_ya_pian,” “zhong_zu_shan_lv_cha”), Essential Hypertension (“yuan_fa_xing_gao_xue_ya”), Primary Hypertension (“yuan_fa_xing_gao_xue_ya”), and randomized (“sui_ ji”). No language restriction was applied.

### Data extraction

Studies were identified according to the inclusion criteria and data were extracted independently by two authors (LWN and YZ). The extracted information included: participant demographics and baseline characteristics; study methodology; details of the intervention and control conditions; outcome measures and main results. Any discrepancies were identified and resolved through discussion with a third author (WJ) if necessary.

### Quality assessment of included studies

Two authors (YXC and YGY) independently assessed the risk of bias using the Cochrane of risk of bias tool (Higgins and Green, 2011). The following items were assessed: Random sequence generation (selection bias); Allocation concealment (selection bias); Blinding (performance bias and detection bias); Incomplete outcome data (attrition bias); Selective outcome reporting (reporting bias).

The risk of bias was categorized as low/unclear/high risk of bias. Trials which met none of the criteria were judged as having a high risk of bias. Trials which met all criteria were judged as having a low risk of bias, and trials with insufficient information to judge were classified as unclear risk of bias. Disagreements between YXC and YGY over the risk of bias in specific studies were resolved by discussion and consensus with involvement of a third review author (WJ). A flow diagram of study selection was generated according to the PRISMA requirements (Moher et al., 2009).

### Criteria for SBP, DBP, and LVM regression

Included studies should report effective improvements of systolic blood pressure or diastolic blood pressure as the major outcome. (1) SBP: systolic blood pressure [e.g., Effective improvements should achieve at least 10 mmHg reduction or normal level (Carretero and Oparil, 2000)]. (2) DBP: diastolic blood pressure [e.g., Effective improvements should achieve at least 10 mmHg reduction (Carretero and Oparil, 2000)]. Participants with essential hypertension regardless of the disease course and severity and diagnosed with any one of the following criteria: (a) The International Classification of Disease (ICD) version 9 or 10; (b) WHO -ISH guidelines for the management of hypertension (WHO-ISH GMH1999), SBP ≥ 160 mmHg, DBP ≥ 95 mmHg; (c) Joint National Committee on the Prevention, Detection, Evaluation, and Treatment of High Blood Pressure (JNC 6), SBP ≥ 140 mmHg, DBP ≥ 90 mmHg; (d) China Guidelines on Prevention and Management of High Blood Pressure (CGPMHBP), SBP ≥ 140 mmHg, DBP ≥ 90 mmHg. We excluded participants with secondary hypertension of other types.

A progressive increase in left ventricular mass (LVM) correlates with exposure to high blood pressure (Verdecchia et al., 1998; Schillaci et al., 2000), so that regression of LVM with pharmacotherapy was reviewed as outcome in our study. According to the American Society of Echocardiography (ASE), the simplified calculation of LVM is LVM = 0.8 (1.04 ([LVIDD + PWTD + IVSTD]^3^− [LVIDD]^3^))+ 0.6 g (Devereux et al., 1986). Left ventricle internal dimension diastole (LVIDd), inter-ventricular septum diastole (IVSd), and posterior wall diastole (PWd) are measured at enddiastole from 2D or M-mode recordings, preferably on several beats. The limits of abnormal ranges for LVM when indexed for body surface area (LVMI) in the ASE chamber quantification are ≥ 120 g/m^2^ in women and ≥ 125 g/m^2^ in men (JNC 6).

### Adverse events

Adverse Events (AEs) of RCTs including non-reported AEs, types and frequency of AEs reported were surveyed for incidence of AEs during EIH treatments. The follow-up were also measured.

## Statistical analysis

We performed meta-analyses using RevMan 5.3 software. Data were summarized by using risk ratios (RR) with 95% confidence intervals (CI) for binary outcomes or mean difference (MD) with 95% CI for continuous outcomes. Fixed effects model was used unless there was evidence of heterogeneity. We assessed heterogeneity using both the Chi-squared test and the I-squared statistic. I-squared statistic was considered if value greater than 25%. For cross-over trials, only the outcomes from the first period were included. We requested data from corresponding author if the required data were not reported. Once outcomes were all evaluated, a summary of findings table was created using the GRADE system (Guyatt et al., 2008).

## Results

### Description of trials

**Figure 2:**
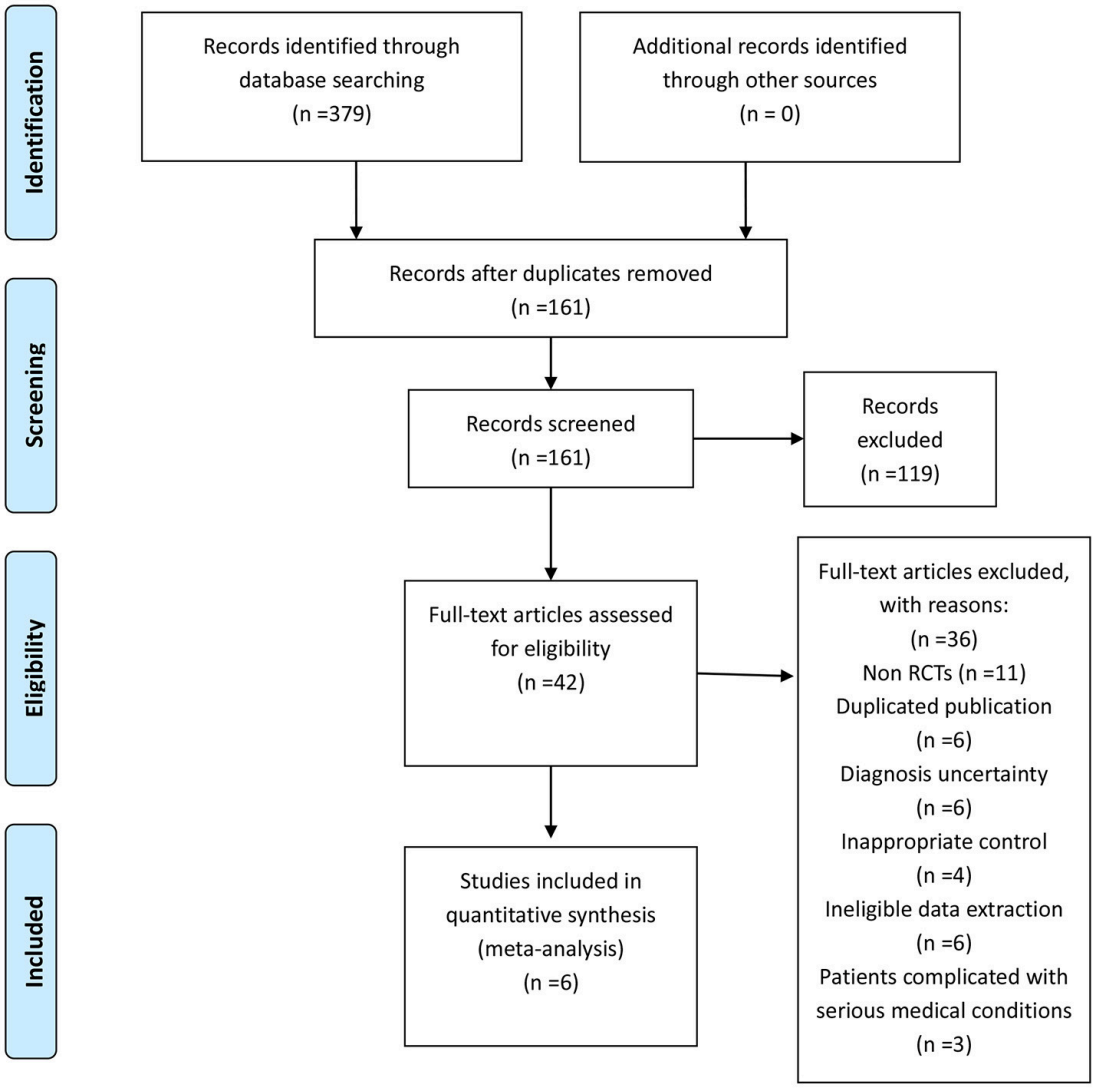
Flow diagram of the literature searching and study selection.

We included 6 RCTs (Yang et al., 2000; Hui and Shu, 2002; Liu, 2002; Tang et al., 2004; Yang, 2006; Su and Guo, 2015) for this systematic review. All RCTs were conducted in China, and all were published in Chinese. A flow chart showed the search process and study selection (Figure 2). Table 1 listed the detailed characteristics of all included trials. The 6 RCTs involved 772 participants with essential hypertension, aged between 41 and 92 years old. The baseline characteristics were listed in Tables 1, 2. About the diagnostic criteria of hypertension used in the included trials, one trials (Yang et al., 2000) used 1999 WHO -ISH guidelines for the management of hypertension (1999 WHO -ISH GMH), one trials (Hui and Shu, 2002) used 1978 WHO -ISH GMH, two trials (Tang et al., 2004; Yang, 2006) used Sixth Report of the Joint National Committee on the Prevention, Detection, Evaluation, and Treatment of High Blood Pressure (JNC 6), two trials (Liu, 2002; Su and Guo, 2015) used China Guidelines on Prevention and Management of High Blood Pressure-2000 (CGPMHBP-2000).

**Table 1:** Characteristics of included trials.

**Study ID**	**Sample size**	**Diagnosis standard**	**Intervention**	**Control**	**Course (week)**	**Outcome measure**
Yang et al., 2000	230	1999 WHO -ISH GMH	EIH (SLCT)	felodipine sustained release tablet	2	BP; side effect
Hui and Shu, 2002	63	1978 WHO -ISH GMH	EIH (SLCT)	captopril	4	BP; LVH; no side effect
Liu, 2002	101	CGPMHBP-2000	EIH (SLCT)	amlodipine	4	BP; no side effect
Tang et al., 2004	80	JNC-VI	EIH (SLCT)	nitrendipine	2	BP; no side effect
Yang, 2006	52	JNC-VI	EIH (SLCT)	captopril	4	BP; LVH; no side effect
Su and Guo, 2015	166	CGPMHBP-2000	EIH (SLCC)	nifedipine	12	BP; side effect

*WHO -ISH GMH, WHO -ISH guidelines for the management of hypertension; CGPMHBP, China Guidelines on Prevention and Management of High Blood Pressure; JNC-VI, Sixth Report of the Joint National Committee on the Prevention, Detection, Evaluation, and Treatment of High Blood Pressure; BP, blood pressure; LVH, left ventricular hypertrophy; EIH, extract of I. hainanensis; SLCT, shan_lv_cha antihypertensive tablet; SLCC, shan_lv_cha antihypertensive capsule. The dosage of EIH varied from 6 to 8 tablets (1.5 to 2 g) per day divided into three times, with an average dose of about 7 tablets (1.75 g) daily*.

**Table 2:** Baseline characteristics.

**Study**	**Intervention**						**Control**					
	***n***	**% Female**	**Duration of disease**	**Mean age (years)**	**Mean SBP (mmHg)**	**Mean DBP (mmHg)**	***n***	**% Female**	**Duration of disease**	**Mean age (years)**	**Mean SBP (mmHg)**	**Mean DBP (mmHg)**
Yang et al., 2000	124	58	9.0 ± 6.3	64.7 ± 8.7	167.7 ± 13.0	105.1 ± 7.9	106	50	10.5 ± 8.5	67.2 ± 8.1	168.9 ± 14.7	106.7 ± 10.3
Hui and Shu, 2002	33	33	51.1 ± 7.8	10.1 ± 5.9	163.8 ± 11.7	101.8 ± 6.3	32	38	9.1 ± 6.4	49.6 ± 9.1	163.8 ± 11.7	101.8 ± 6.3
Liu, 2002	49	57	?	41~71^#^	167.5 ± 7.2	100.6 ± 5.6	52	54	?	45~69^#^	168.6 ± 9.1	105.2 ± 6.1
Tang et al., 2004	80	40	14.2 ± 4.2	63 ± 6	?>140	?>90	80	35	13.6 ± 4.6	64 ± 5	?>140	?>90
Yang, 2006	30	36	8~26^#^	60 ± 15.3	165.73 ± 21.92	103.22 ± 11.45	30	30	5~24^#^	64 ± 19.1	168.67 ± 23.62	105.21 ± 10.37
Su and Guo, 2015	83	53	6.2 ± 1.1	64.6 ± 5.3	150.2 ± 23.1	95.1 ± 13.2	83	53	6.2 ± 1.1	64.6 ± 5.3	153.7 ± 24.5	93.6 ± 11.7

# *The range of age or year is given. Mean values are not given. Standard deviation is not given*.

? *The range of age or year is not given. Mean values are not given. Standard deviation is not given*.

There were two main comparisons: EIH vs. conventional antihypertensive medicine (5 trials Yang et al., 2000; Hui and Shu, 2002; Liu, 2002; Tang et al., 2004; Su and Guo, 2015, 83%), and EIH plus conventional antihypertensive medicine vs. conventional antihypertensive medicine (1 trial Yang, 2006, 17%). The conventional antihypertensive medicines included felodipine sustained-release tablet, captopril, amlodipine, nitrendipine, and nifedipine. The duration of treatment varied from 2 to 12 weeks, with an average duration of 4.7 weeks. The dosage of EIH varied from 6 to 8 tablets (1.5–2 g) per day divided into three times, with an average dose of about 7 tablets (1.75 g) daily. All ingredients in the medication were in equal proportion across different manufacturing products.

### Risk of bias assessment

**Figure 3:**
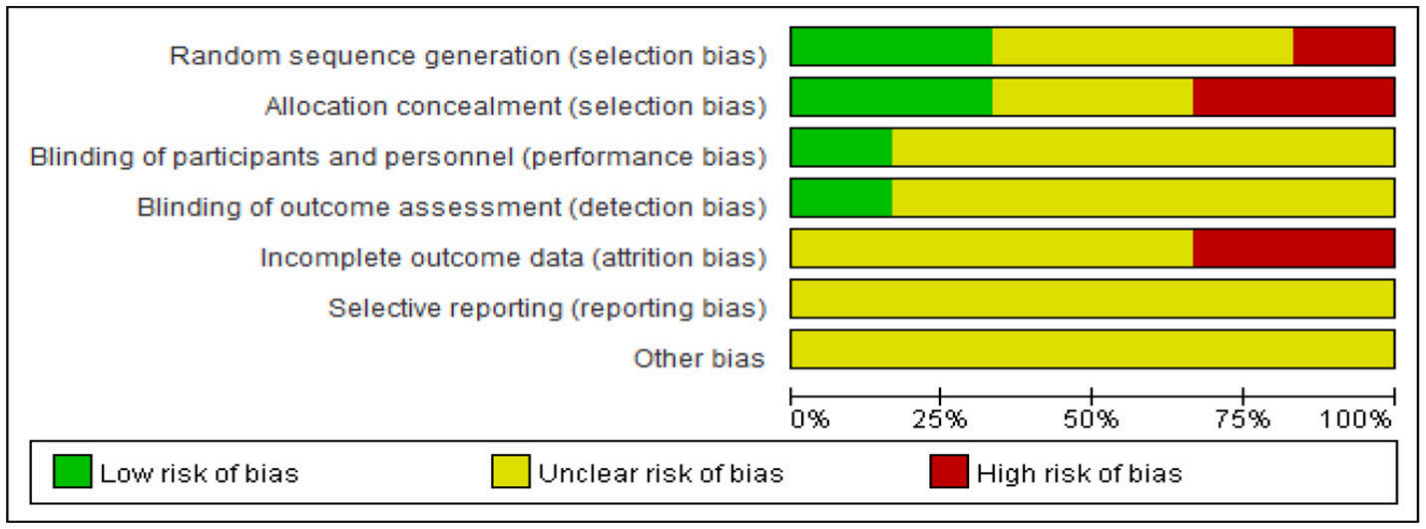
Risk of bias graph: review authors' judgments about each risk of bias item presented as percentages across all included studies.

The risk of bias were assessed include random sequence generation, allocation concealment, blinding, incomplete outcome data, selective outcome reporting. For random sequence generation, one trials (17%) (Hui and Shu, 2002) used a random number table, one trial (17%) (Yang, 2006) used treatment sequence, and the other four trials (66%) just simply mentioned “randomization” and did not report the specific method of random sequence generation. For allocation concealment, two trial (33%) (Liu, 2002; Yang, 2006) used a center controlled method, while the other four trials (67%) did not report information on this. For blinding, one trial (17%) (Su and Guo, 2015) used single-blinding method in which just mentioned “single blinding,', but did not give detailed information. The other five trials (83%) did not give information on blinding. For incomplete outcome data, only one trial (17%) (Yang, 2006) reported the detailed information of attrition by describing the number and reasons for withdrawal. However, it was still unclear whether there was incomplete outcome data in those trials, for the number of participants in randomization and in data analysis was not equal. For selective outcome reporting, all trials did not report the information of registration. We could not make a comparison between the protocols and trial reports. In conclusion, after assessing the risk of bias the 6 included trials, we found the general methodological quality of most trials was low (Figure 3, Table 3). Using the outcomes reviewed, a summary of findings table has been created using the GRADE system (Table 4). The three most relevant patient important outcomes are displayed in the table.

**Table 3:** Methodological quality of included studies based on the Cochrane handbook.

**Included trials**	**A**	**B**	**C**	**D**	**E**	**F**
Yang et al., 2000	−	−	**?**	**?**	−	**?**
Hui and Shu, 2002	+	**?**	**?**	**?**	**?**	**?**
Liu, 2002	**?**	+	**?**	**?**	**?**	**?**
Tang et al., 2004	**?**	−	**?**	**?**	−	**?**
Yang, 2006	+	+	**?**	**?**	**?**	**?**
Su and Guo, 2015	**?**	**?**	+	+	**?**	**?**

*A, random sequence generation; B, allocation concealment; C, blinding of participants and personnel; D, blinding of outcome assessment; E, incomplete outcome data; F, selective reporting;+, low risk; −, high risk; ?, unclear*.

**Table 4:** Summary of findings table: the three most important patients outcomes are listed in the summary of findings table.

**4.1 EIH vs. Antihypertensive drugs**					
**Patients or population: patients with essential hypertension**					
**Settings: in adult patients**					
**Intervention: EIH**					
**Outcomes**	Illustrative comparative risks^*^ (95% CI)		**No of Participants (studies)**	**Quality of the evidence (GRADE)**	**Comments**
	**Assumed risk control**	**Corresponding risk EIH**			
Systolic blood pressure	The mean systolic blood pressure ranged across control groups from 134.24 to 151.2 mmHg	The mean systolic blood pressure in the intervention groups was 0 higher (2.3 lower to 1.43 higher)	606(5 studies)	⊕⊕⊖⊖low	
Diastolic blood pressure	The mean distolic blood pressure ranged across control groups from 84.4 to 92.4 mmHg	The mean diastolic blood pressure in the intervention groups was 0.02 lower(1.13 lower to 1.09 higher)	606(5 studies)	⊕⊕⊖⊖low	
Left ventricular mass index	The mean left ventricular mass index ranged across control groups from 91.41 to 122.2 g/m^2^	The mean left ventricular mass index in the intervention groups was 1.36 lower(4.99 lower to 2.26 higher)	85(2 studies)	⊕⊕⊖⊖low	
**4.2 EIH plus Antihypertensive drugs vs. Antihypertensive drugs**					
**Patients or population: patients with essential hypertension**					
**Settings: in adult patients**					
**Intervention: EIH plus Antihypertensive drugs**					
Systolic blood pressure	The mean systolic blood pressure of the control group in single study was 131.8 mmHg	The mean systolic blood pressure in the intervention group was 8.5 lower(11.99 to 5.01 lower)	166(1 study)	⊕⊕⊖⊖low	
Diastolic blood pressure	The mean diastolic blood pressure of the control group in single study was 82.5 mmHg	The mean diastolic blood pressure in the intervention group was 8.7 lower(11.49 to 5.91 lower)	166(1 study)	⊕⊕⊖⊖low	
GRADE Working Group grades of evidence					
High quality: Further research is very unlikely to change our confidence in the estimate of effect.					
Moderate quality: Further research is likely to have an important impact on our confidence in the estimate of effect and may change the estimate.					
Low quality: Further research is very likely to have an important impact on our confidence in the estimate of effect and is likely to change the estimate.					
Very low quality: We are very uncertain about the estimate.					

* *The basis for the assumed risk (e.g., the median control group risk across studies) is provided in footnotes. The corresponding risk (and its 95% confidence interval) is based on the assumed risk in the comparison group and the relative effect of the intervention (and its 95% CI)*.

*CI: Confidence interval*.

### Effect of the interventions

#### Systolic blood pressure

**Figure 4:**
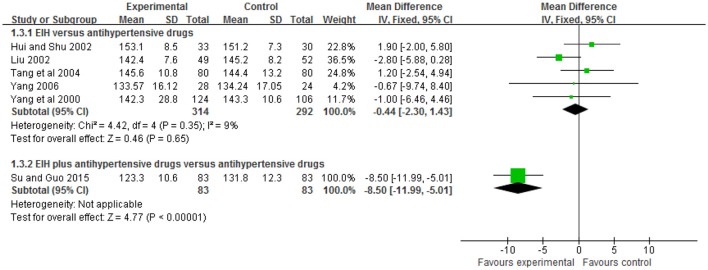
The forest plot of comparison of two groups for the outcome of systolic blood pressure.

Five trials (Yang et al., 2000; Hui and Shu, 2002; Liu, 2002; Tang et al., 2004; Yang, 2006) compared the preparation of EIH used alone with antihypertensive drugs. There was no significant difference between the two groups in systolic blood pressure (WMD: −0.44 [−2.30, 1.43]; *P* = 0.65); one trial (Su and Guo, 2015) compared the preparation of EIH plus antihypertensive drugs with antihypertensive drugs. There was significant difference between the two groups in systolic blood pressure (WMD: −8.50 [−11.99, −5.01]; *P* < 0.00001) (see Figure 4, Table 5).

**Table 5:** Analyses of systolic blood pressure.

**Trials**		**WMD [95% CI]**
**EIH vs. ANTIHYPERTENSIVE DRUGS**		
Shan_lv_cha tablet vs. captopril	1	1.90 [−2.00, 5.80]
Shan_lv_cha tablet vs. amlodipine	1	−2.80 [−5.88, 0.28]
Shan_lv_cha tablet vs. nitrendipine	1	1.20 [−2.54, 4.94]
Shan_lv_cha tablet vs. captopril	1	−0.67 [−9.74, 8.40]
Shan_lv_cha tablet vs. felodipine sustained release tablet	1	−1.00 [−6.46, 4.46]
Meta-Analysis	5	−0.44 [−2.30, 1.43]
**EIH PLUS ANTIHYPERTENSIVE DRUGS vs. ANTIHYPERTENSIVE DRUGS**		
Shan_lv_cha capsule plus nifedipine vs. nifedipine	1	−8.50 [−11.99, −5.01]
Meta-Analysis	1	−8.50 [−11.99, −5.01]

*EIH, extract of I. hainanensis*.

#### Diastolic blood pressure

**Figure 5:**
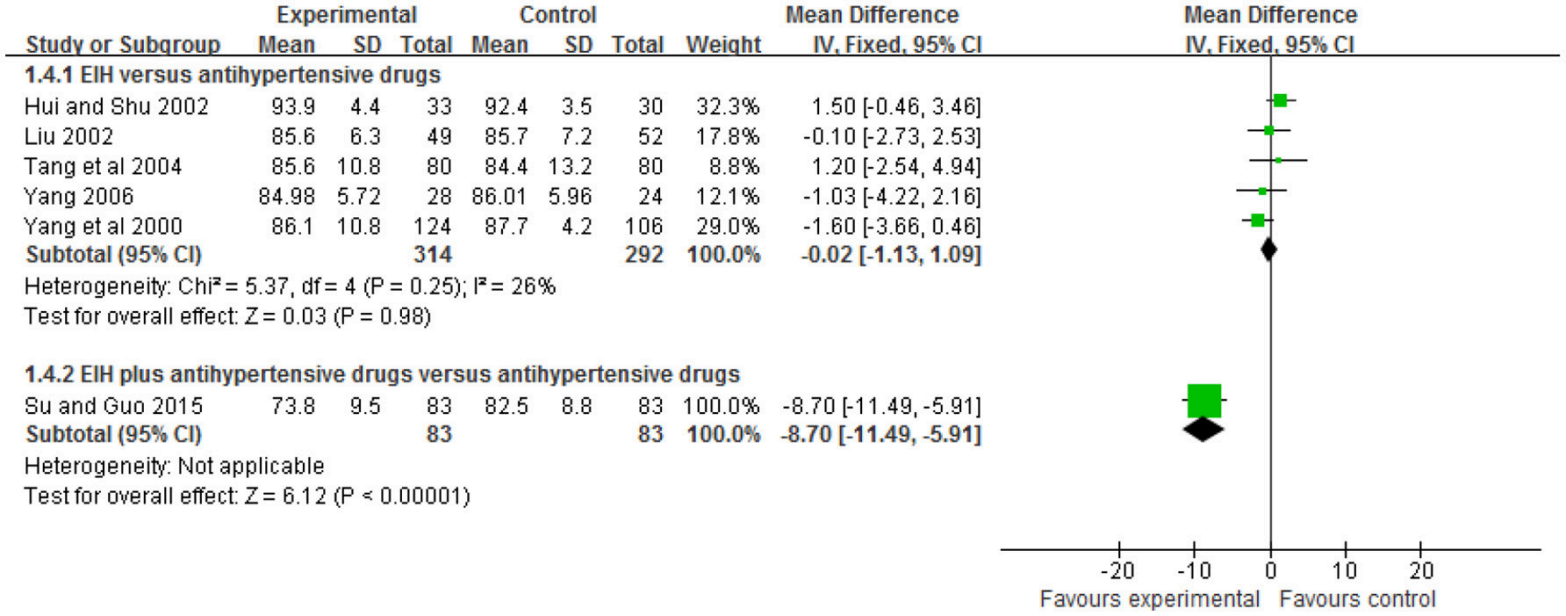
The forest plot of comparison of two groups for the outcome of diastolic blood pressure.

Five trials (Yang et al., 2000; Hui and Shu, 2002; Liu, 2002; Tang et al., 2004; Yang, 2006) compared the preparation of EIH used alone with antihypertensive drugs. There was no significant difference between the two groups in diastolic blood pressure (WMD: WMD: −0.02 [−1.13, 1.09]; *P* = 0.98); and one trial (Su and Guo, 2015) compared the preparation of EIH plus antihypertensive drugs with antihypertensive drugs. There was significant difference between the two groups in systolic blood pressure (WMD: −8.70 [−11.49, −5.91]; *P* < 0.00001) (see Figure 5, Table 6).

**Table 6:** Analyses of diastolic blood pressure.

**Trials**		**WMD [95% CI]**
**EIH vs. ANTIHYPERTENSIVE DRUGS**		
Shan_lv_cha tablet vs. captopril	1	1.50 [0.46, 3.46]
Shan_lv_cha tablet vs. amlodipine	1	−0.10 [−2.73, 2.53]
Shan_lv_cha tablet vs. nitrendipine	1	1.20 [−2.54, 4.94]
Shan_lv_cha tablet vs. captopril	1	−1.03 [−4.22, 2.16]
Shan_lv_cha tablet vs. felodipine sustained release tablet	1	−1.60 [−3.66, 0.46]
Meta-Analysis	5	−0.02[−1.13, 1.09]
**EIH PLUS ANTIHYPERTENSIVE DRUGS vs. ANTIHYPERTENSIVE DRUGS**		
Shan_lv_cha capsule plus nifedipine vs. nifedipine	1	−8.70 [−11.49, −5.91]
Meta-Analysis	1	−8.70 [−11.49, −5.91]

*EIH, extract of I. hainanensis*.

#### Regression of left ventricular mass

**Figure 6:**
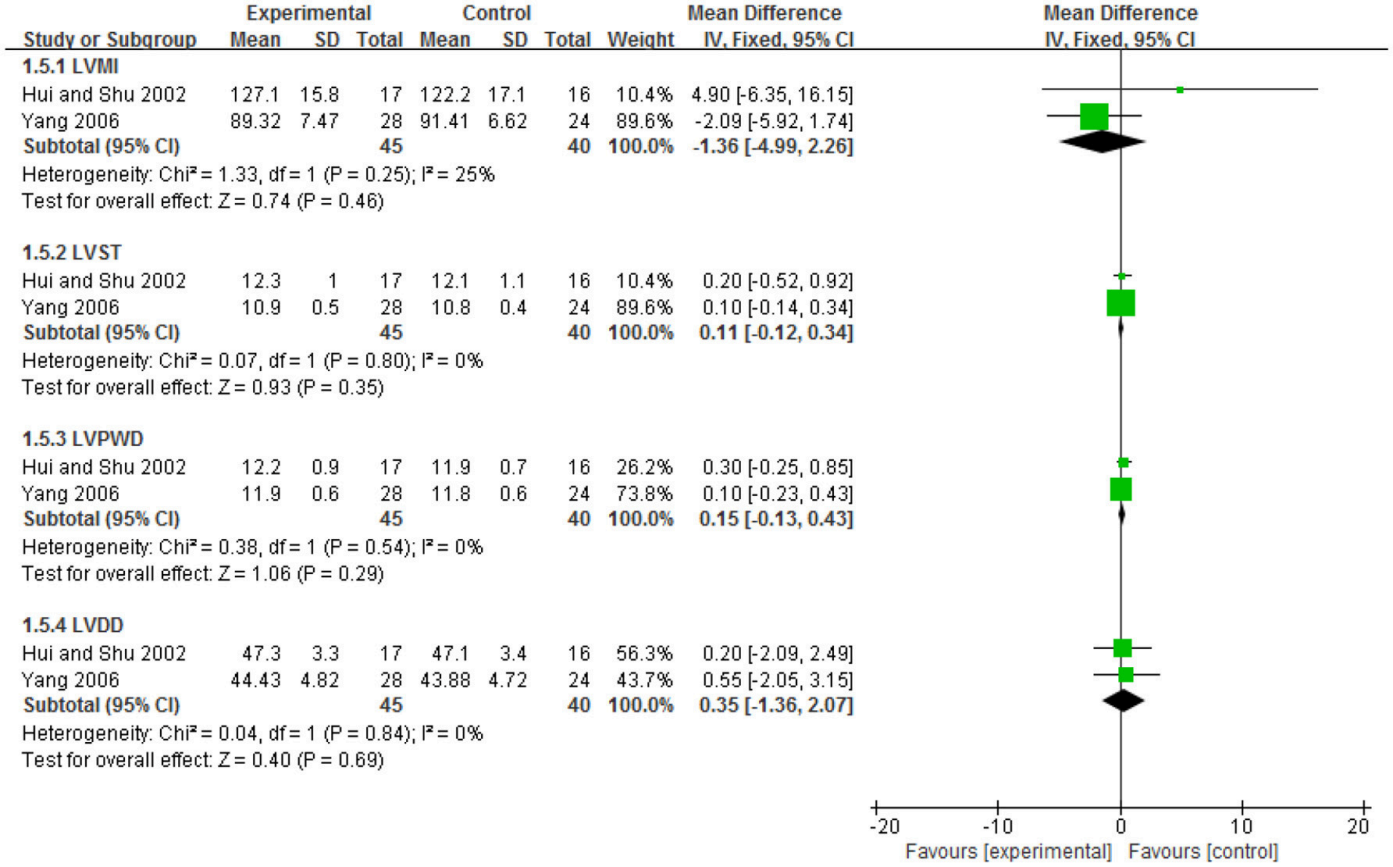
The forest plot of comparison of two groups for the outcome of left ventricular mass.

Two trials (Hui and Shu, 2002; Yang, 2006) compared the preparation of EIH used alone with antihypertensive drugs. There was no significant difference between the two groups in LVMI (WMD: −1.36 [−4.99, 2.26]; *P* = 0.46), LVST (WMD: 0.11 [−0.12, 0.34]; *P* = 0.35), LVPWD (WMD: 0.15 [−0.13, 0.43]; *P* = 0.29), LVDD (WMD: 0.35 [−1.36, 2.07]; *P* = 0.69). Both of the two trials (Hui and Shu, 2002; Yang, 2006) showed that after 4 weeks of treatment, the level of LVMI in both EIH group and captopril group decreased significantly (*P* < 0.05), but there was no significant difference between these two group (*P* = 0.46) (see Figure 6, Table 7).

**Table 7:** Analyses of left ventricular mass.

**Trials**		**WMD [95% CI]**
**EIH vs. ANTIHYPERTENSIVE DRUGS: LVMI**		
Shan_lv_cha tablet vs. captopril	1	4.90 [−6.35, 16.15]
Shan_lv_cha tablet vs. captopril	1	−2.09 [−5.92, 1.74]
Meta-Analysis	2	−1.36[−4.99, 2.26]
**EIH vs. ANTIHYPERTENSIVE DRUGS: LVST**		
Shan_lv_cha tablet vs. captopril	1	0.20 [−0.52, 0.92]
Shan_lv_cha tablet vs. captopril	1	0.10 [−0.14, 0.34]
Meta-Analysis	2	0.11 [−0.12, 0.34]
**EIH vs. ANTIHYPERTENSIVE DRUGS: LVPWD**		
Shan_lv_cha tablet vs. captopril	1	0.30 [−0.25, 0.85]
Shan_lv_cha tablet vs. captopril	1	0.10 [−0.23, 0.43]
Meta-Analysis	2	0.15 [−0.13, 0.43]
**EIH vs. ANTIHYPERTENSIVE DRUGS: LVDD**		
Shan_lv_cha tablet vs. captopril	1	0.20 [−2.09, 2.49]
Shan_lv_cha tablet vs. captopril	1	0.55 [−2.05, 3.15]
Meta-Analysis	2	0.35 [−1.36, 2.07]

*EIH, extract of I. hainanensis*.

### Side effect

Two include trials (Yang et al., 2000; Su and Guo, 2015) mentioned the side effects in EIH group. The adverse effects only included diarrhea 0.25% (1/397), nausea 0.25% (1/397), peripheral edema 0.25% (1/397), hypotension 0.25% (1/397), and elevation of aminotransferase 0.25% (1/397). No severe adverse events were reported. These adverse effects disappeared after continue to take medicine. The other four trials (Hui and Shu, 2002; Liu, 2002; Tang et al., 2004; Yang, 2006) reported no side effect in EIH group compared to conventional antihypertensive drugs.

### Sensitivity analysis and publication bias

We failed to conduct sensitivity analysis, and also failed to perform funnel plot to detect publication bias, because of no sufficient number of trials.

## Discussion

In our review, five trials (Yang et al., 2000; Hui and Shu, 2002; Liu, 2002; Tang et al., 2004; Yang, 2006) compared the preparation of EIH used alone with antihypertensive drugs. There was no significant difference between the two groups in systolic and diastolic blood pressure; however, one trial (Su and Guo, 2015) compared the preparation of EIH plus antihypertensive drugs with antihypertensive drugs. There was statistically significant difference between the two groups in systolic and diastolic blood pressure (*P* < 0.00001). As a progressive increase in left ventricular mass (LVM) correlates with exposure to high blood pressure (Verdecchia et al., 1998; Schillaci et al., 2000), the regression of LVM with pharmacotherapy was also included as outcome in our study. Two trials (Hui and Shu, 2002; Yang, 2006) showed that after 4 weeks of treatment, the level of LVMI in both EIH group and captopril group decreased significantly (*P* < 0.05), but there was no significant difference between these two group (*P* = 0.46). After all, four trials (Yang et al., 2000; Liu, 2002; Tang et al., 2004; Su and Guo, 2015) were compared with calcium channel blockers (nitrendipine, amlodipine, and felodipine, nifedipine) and two trials (Hui and Shu, 2002; Yang, 2006) compared with ACE inhibitors (captopril). There was no evidence for the effect of EIH compared with other antihypertensive drug classes such as beta-blockers, angiotensin II receptor antagonists, and diuretics. As noted that ACE inhibitors decrease left ventricular mass more effectively than beta-blockers and its mechanism is related to activation of the renin-angiotensin-aldosterone system, in particular increased angiotensin II levels, and stimulating growth of myocardial cells (Nagano et al., 1991). The findings were limited by the small sample sizes, duration and low methodological quality of the trials. More rigorous RCTs comparing EIH with ACE inhibitors or other antihypertensive drug classes are urgently needed.

Though this review suggested some benefit of *Ilex hainanensis Merr*. preparation for essential hypertension, the following problems contribute to the limited methodological quality of most included trials: (a) For blinding, who was blinded was unclear; (b) For random sequence generation and allocation concealment, most were unclear; (c) For incomplete outcome data and selective outcome reporting, dropout during the trial and use of intention-to-treat analysis was unclear; Using the outcomes reviewed, a summary of findings table has been created using the GRADE system (Table 6). The three most relevant patient important outcomes are displayed in the table. We highly recommend further RCT should report according to the CONSORT Statement (Schulz et al., 2010; Wang et al., 2013, 2014; Yang et al., 2015).

A total of 6 databases had been searched up to Jan 2018 including the Cochrane library, PubMed, Medline, Wanfang, CNKI, and VIP. It might have a limitation of missing some trials since we are unable to search pertinent databases such as Allied and Alternative medicine (AMED) and Excerpt Medica Database (EMBASE). All studies were mainly published in Chinese, and the strength of the evidence was limited by the lack of placebos. 6 RCTs and a total of 772 participants were included. No trials claimed “negative” effect of *Ilex hainanensis Merr*. Prospective registration of clinical trials and/or publication of clinical trial protocol may be a good solution (Xu and Chen, 2008; Robinson, 2011).

Though none of the included trials reported severe adverse events possibly related to *Ilex hainanensis Merr*., we cannot draw firm conclusions about the safety of *Ilex hainanensis Merr*. because of insufficient trials. The EIH has been widely used in three preparations: shan_lv_cha antihypertensive capsule, jue_ming_shan_lv_cha tablet, and shan_lv_cha antihypertensive tablet, which has been included in the Pharmacopeia of the People's Republic of China in 2010. The dosage of EIH varied from 6 to 8 tablets (1.5–2 g) per day divided into three times, with an average dose of about 7 tablets (1.75 g) daily. Two trial (Hui and Shu, 2002; Yang, 2006) mentioned that total 10 patients withdrew (2 cases in EIH group, 8 cases in antihypertensive drugs group) because of unsatisfied blood pressure (SBP/DBP>160/95 mmHg). The percentage of missing data is 1%. The duration of treatment of trials ranged from 2 to 12 weeks, so the potential beneficial or harmful effect of *Ilex hainanensis Merr*. for treatment of essential hypertension might only result from short treatment duration.

With the acceptance and popularity of TCM, great contributions have been made to the health and well-being of the people for the unique advantages of TCM in cardiovascular disease (Hao et al., 2015, 2017). *Ilex hainanensis Merr*. is used as an agent for resolving edema and relieving pain, invigorating blood and unblocking the collaterals in TCM. The antihypertensive effect is one of the main pharmacological effects of *Ilex hainanensis* Merr. Since most participants with essential hypertension require long-time treatment, the long-term safety of the treatment is still an important concern. We recommend that further studies should pay attention to the adverse events and long-term safety by designing a longer duration of treatment and a long-term follow-up (Adriane, 2000; Windrum et al., 2000; Tachjian et al., 2010). In future, more rigorous RCTs and double blind randomized placebo controlled clinical trials of long term duration measuring mortality and cardiovascular morbidity as well as blood pressure are urgently needed.

## Conclusion

The extract of *Ilex hainanensis Merr*. and its preparations seems to have some beneficial effects on lowering systolic and diastolic blood pressure, left ventricular mass in participants with essential hypertension. However, the findings were limited by the small sample sizes, duration and low methodological quality of the trials. Further rigorously designed, and well reported RCTs are still needed to prove the effectiveness and safety of the extract of *Ilex hainanensis Merr*. and its preparations for essential hypertension.

## Author contributions

XY conceived and designed this meta-analysis and wrote the paper. XY and GY collected the data and drew the tables. YZ, WL, and JW analyzed the data and helped to draft the manuscript together.

### Conflict of interest statement

The authors declare that the research was conducted in the absence of any commercial or financial relationships that could be construed as a potential conflict of interest. The reviewer VM and handling Editor declared their shared affiliation.
